# Treating Children With Advanced Rheumatic Heart Disease in Sub-Saharan Africa: The NGO EMERGENCY's Project at the *Salam* Centre for Cardiac Surgery in Sudan

**DOI:** 10.3389/fped.2021.704729

**Published:** 2021-08-20

**Authors:** Rossella Miccio, Maria Quattrociocchi, Lorenzo Valgoi, Liliane Chatenoud, Salvatore Lentini, Elena Giovanella, Luca Rolla, Nicoletta Erba, Sofia Gatti, Daniela Rocchi, Manahel Badr Saad, Alessandro Salvati, Martin Langer, Gina Portella, Gino Strada

**Affiliations:** ^1^Emergency ONG ONLUS, Milan, Italy; ^2^Department of Public Health, Laboratory of Clinical Epidemiology, Istituto di Ricerche Farmacologiche Mario Negri IRCCS, Milan, Italy; ^3^Federazione Centri per la Diagnosi Della Trombosi e la Sorveglianza delle Terapie Antitrombotiche (FCSA), Milan, Italy; ^4^Salam Centre for Cardiac Surgery, Emergency ONG ONLUS, Khartoum, Sudan

**Keywords:** rheumatic heart disease, cardiac surgery, children, Sudan, Sub-Saharan Africa, EMERGENCY ONG Onlus, humanitarian surgery

## Abstract

Rheumatic heart disease is endemic in Sub-Saharan Africa and while efforts are under way to boost prophylaxis and early diagnosis, access to cardiac surgery is rarely affordable. In this article, we report on a humanitarian project by the NGO EMERGENCY, to build and run the *Salam* Centre for Cardiac Surgery in Sudan. This hospital is a center of excellence offering free-of-charge, high-quality treatment to patients needing open-heart surgery for advanced rheumatic and congenital heart disease. Since it opened in 2007, more than 8,000 patients have undergone surgery there; most of them Sudanese, but ~20% were admitted from other countries, an example of inter-African cooperation. The program is not limited to surgical procedures. It guarantees long-term follow-up and anticoagulant treatment, where necessary. By way of example, we report clinical features and outcome data for the pediatric cohort: 1,318 children under the age of 15, operated on for advanced rheumatic heart disease between 2007 and 2019. The overall 5-year survival rate was 85.0% (95% CI 82.7–87.3). The outcomes for patients with mitral valves repaired and with mitral valves replaced are not statistically different. Nevertheless, observing the trend of patients undergoing valve repair, a better outcome for this category might be assumed. RHD in children is an indicator of poor socio-economic conditions and an inadequate health system, which clearly will not be cured by cardiac surgery alone. Nevertheless, the results achieved by EMERGENCY, with the crucial involvement and participation of the Sudanese government over the years, show that building a hospital, introducing free cardiac surgery, and offering long-term post-operative care may help spread belief in positive change in the future.

## Introduction

Rheumatic heart disease (RHD) is a disease connected to poverty: group A streptococcal infection is the first event, which may be followed in sequence by acute rheumatic fever (ARF) and RHD. Streptococcal exposure due to poor, overcrowded housing, poor hygiene in countries with poor access to primary healthcare and limited opportunities for treatment, are the main risk factors, probably dominant over difference in susceptibility (1, 2). RHD results from this not fully understood immunological process and remains a scourge among young adults and children in low- and middle-income countries (LMICs) (3–5). It is well-known that this form of heart disease is preventable in individuals and can be eradicated in a population, as has happened in high-income countries in the last century (6).

In Sudan, the prevalence of RHD is reported to be 61 per 1,000 people (7). Prevalence values are highly dependent on the sensitivity of the screening method and show regional differences (8). Thanks to lower susceptibility, better treatment and effective primary and secondary prophylaxis, many patients will not progress to severe, symptomatic RHD and its complications (heart failure, endocarditis, thromboembolism, and stroke). However, there are no other non-invasive therapeutic options in case of progression, and open-heart surgery with valve repair or replacement becomes life-saving when prevention has failed (6).

The estimated need for cardiac surgery for RHD in endemic countries in Sub-Saharan Africa (SSA) is around 150 per year per million people (9). This number is far from manageable, and only about 5% of patients with severe RHD had access to heart surgery (10).

Many calls for action have come from African and international organizations with a view to intensifying global attempts to reduce the burden of rheumatic heart disease (9–16). They raised the issue of RHD as a neglected non-communicable disease, as well as the lack of cardiac surgery for patients with advanced RHD in LMICs. Over the last 25 years, the mortality reduction in this part of Africa has been much less evident than in other parts of the world where RHD is endemic (17). However, specific national programs are now established, and echocardiographic screening and earlier diagnoses will lead to earlier recognition (7) and improved prophylaxis. Unfortunately, cardiac surgery as rescue treatment for symptomatic RHD patients will continue to be necessary for a long time as the only life-saving treatment.

How has the availability of cardiac surgery been in SSA over the last 20 years? The survey published by Yanakah et al. (18) in 2012 reports on 22 cardiac centers in SSA, with 1,277 procedures in the previous year, many of them performed by visiting teams. The same authors identified and reviewed three models for developing cardiac surgery in the region. A recent paper by Murala et al. (10) analyzes the problem of cardiac surgery for CHD in SSA and reports a long list of groups performing cardiac surgical missions. Affordability and accessibility remain minimal, however.

In this contribution to the topic of “Pediatric Cardiology and Cardiac Surgery in Developing Countries,” we will try to present as a model the project of the Centre for Cardiac Surgery in Sudan, built and run by the NGO EMERGENCY with the support of the Sudanese government. Its organizational strengths will be assessed by reporting on one of the patient groups, children operated on for RDH.

### EMERGENCY–NGO (https://en.emergency.it/what-we-do/healthcare-.excellence-in-africa/)

When EMERGENCY was founded in 1994, its main goal was to provide life-saving surgery for war-wounded people by building and running hospitals in some of the countries most covered in landmines and devastated by conflicts. We knew that nine times out of 10, war-wounded people are civilians, not combatants, but over the years, we also learned that victims of war are not just people injured and maimed by shrapnel, bullets, and landmines. Victims of war are also all the men, women and children whose right to access healthcare is neglected because fighting has destroyed all health infrastructure. Our organization's long experience in dealing with these contexts has taught us that treating urgent medical needs is essential, but the only preventive to war is building up human rights and overcoming inequalities.

The idea of establishing a center for cardiac surgery in Africa originated from the belief that healthcare is a fundamental human right and that it must be free of charge and of a high standard for every individual, without discrimination. In the summer of 2003, the Federal Ministry of Health of Sudan, the country affected by the longest civil war in Africa, invited EMERGENCY's representatives to evaluate possible forms of cooperation to support its population. The delegation discussed with the local authorities the possibility of establishing a center of excellence, to provide free-of-charge, high-quality cardiac surgery to patients from Sudan and nine neighboring countries, and also to train local health workers.

At that time, no significant attention was paid to non-communicable diseases, particularly not RHD, so no reliable data was available to assess the real need in the continent or in the country. Nonetheless, our proposal was well-received by the Sudanese authorities because it came from a different standpoint: the aim was to share with African citizens the best achievements of medical science in the name of equality in dignity and rights, as stated in the Universal Declaration of Human Rights. Moreover, at that time, Sudan was the country with the largest number of neighboring states, the perfect place to prove that medicine can be a means of cooperation that overcomes political difficulties. Finally, we believed that excellence could have positive effects on the entire health system, demanding transparent and efficient management, allowing effective in-country training, increasing medical knowledge, and improving quality standards.

### *Salam* Centre for Cardiac Surgery

The *Salam* Centre is a hospital for cardiac surgery; it has three operating theaters (OTs) fully equipped for open-heart cardiac surgery, a 15-bed intensive care unit (ICU), 48 sub-intensive care and ward beds, a laboratory, a radiological department with computer tomography and a cath lab. The outpatient department includes consultation rooms, ultrasound labs, and the oral anticoagulant clinic. Physiotherapy, pharmacy, and a blood bank are active. A guesthouse hosts patients that do not live near the facility during their first month after discharge from hospital. The center has an autonomous electric power supply and all the necessary support services, including a residential compound presently housing the 50–60 members of the foreign team (physicians, nurses, technicians, administrative, and technical staff). The local health staff include ~90 nurses, 50 technicians, pharmacists, physiotherapists, and biomedical engineers.

The *Salam* Centre is also involved in training healthcare workers like nurses, laboratory and X-ray technicians, and medical officers (1-year rotation program). Recently, the center started cooperating with the Sudanese Medical Specialization Board on a program for residents in cardiac surgery, anesthesiology, cardiology, and ICU nursing (Supplementary Table 1 lists all the participants in the working group who have followed one another over the years).

### Oral Anticoagulant Clinic

Valve surgery with implantation of mechanical heart valves is by far the most frequent procedure at the *Salam* Centre, and the resulting need for adequate anticoagulant treatment led to the establishment of the OAC. This activity is run in cooperation with Federazione Centri per la Diagnosi della Trombosi e la Sorveglianza delle Terapie Antitrombotiche (FCSA) and is based on education of patients, free tests, drug supply, and counseling whenever necessary. Before surgery, the local staff give patients – or their parents in the case of children – information about the importance of this treatment, its life-long duration and the necessity of checking the prothrombin time expressed as the international normalized ratio (INR). After surgery, patients return to the OAC for more face-to-face counseling and written instructions. Patients living in Khartoum or the surrounding area come to the *Salam* Centre for their INR check. Patients living in remote areas have their INR taken at local laboratories and tell the OAC their results either over the phone or on WhatsApp, so they can receive their prescription.

Trained receptionists, nurses, local doctors and pharmacists, speaking Arabic as well as English, do the daily work in the OAC, and a foreign expert doctor supervises the work for some months every year. The monitoring of oral anticoagulant therapy (OAT) is supported by dedicated software (PARMA GTS®, Werfen, Milan). Patient demographics and clinical data are recorded in the program, which allows for reporting and provides the physician in charge with suggestions for OAT dose prescriptions thanks to a dose calculation algorithm that is validated beforehand.

Patients are given triage every time they arrive, asking them for their previous warfarin dose report and about the clinical conditions and their compliance with the therapeutic program. The same rules apply for patients giving their INR results over the phone or on WhatsApp. Based on test results and clinical data, the new warfarin program and the next INR control date are established, and a written report and the necessary drug supply are given to outpatients; the “phone call patients” receive their dose prescription by phone the same day; WhatsApp patients receive a PDF prescription on WhatsApp. Remote patients with regular INR checks receive a drug supply every 2 months.

In case of adverse events, such as INR <1.5 or >5, or delays of over 7 days for their agreed check, patients will receive advice from a doctor. If their INR is 0.5 points lower than the lower limit of the therapeutic range, a bridge therapy with full doses of LMWH is added for 2 days.

To avoid losing contact with them, patients who do not come for their INR check receive reminder calls after 2 weeks, 1 month, and 3 months. Patients are considered “lost to follow-up” when no INR test is available for 6 months.

Every thrombotic event (TIA, stroke, embolism, and valve blockage) and major bleeding, classified according to ISTH criteria, as well as deaths, are recorded in the database.

The number of children recorded in the PARMA database is currently (March 2021) 1,195 out of 8,101 patients, 1,042 of them with mechanical heart valves, 115 with biological valves, and 24 non-surgical patients. Out of the children with mechanical valves, 61% are on OAT monitoring at the *Salam* Centre, 14% died, 25% are abroad or lost to follow-up. A detailed analysis of the quality of the OAT treatment will be published soon.

### Regional Program

**Figure 1:**
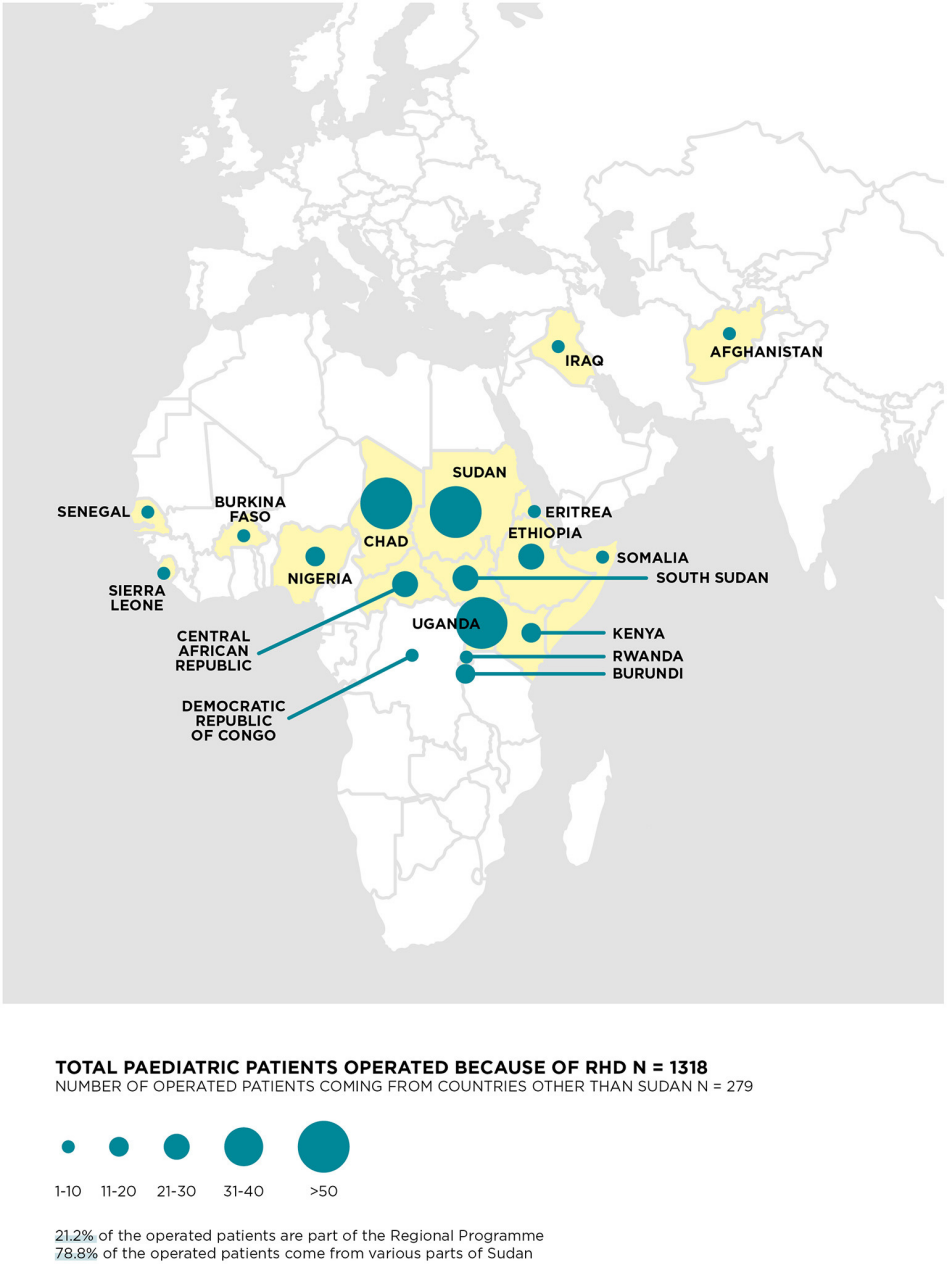
Countries of origin of the 1,318 RHD children.

Over the years, staff at the *Salam* Centre have operated on patients from 32 different countries (Figure 1). To this end, EMERGENCY signed a memorandum of understanding with the Sudanese Ministry of Health to formalize patient referrals. Agreements with local partners, universities, public and private hospitals, and humanitarian organizations make this cooperation possible and effective.

EMERGENCY's international cardiologists carry out regular missions to identify patients in need of referral to surgery and perform follow-up care on patients who have already had their operation. The program manager organizes the missions with the support of local authorities and health workers, who will take care of the patients after returning home and stay in contact with the *Salam* Centre. Many patients are children and travel in groups; the travel expenses are covered mainly by EMERGENCY and sometimes by other humanitarian organizations. The Sudanese government offers free entry visas in recognition of the importance of the project and the role of the *Salam* Centre. Before and after their hospitalization, patients are hosted at the guesthouse in the hospital complex for assessment and convalescence. After discharge, EMERGENCY assures the delivery of the necessary drugs to their home countries and supports anticoagulant therapy remotely.

Some numbers can best explain the activity in the Regional Program: taking the year 2019, there were 10 missions by cardiologists on which 1,220 patients were assessed (630 of these were follow-up visits, 590 as potential candidates for cardiac surgery). In the same year, 159 patients were transferred and operated on at the *Salam* Centre; 75 were children (34 RHD, 41 CHD), 85 of them 15 or over (81 RHD, 3 CHD).

Everybody at EMERGENCY is well aware that all these complex interventions are just drops in the ocean. Still, they can pave the way for a new model of cooperation between African countries as well as between them and humanitarian organizations.

## Valve Surgery in Children Suffering from RHD (2007–2019)

This article, by way of example of the work at the centers, reports on the pediatric RHD population (under the age of 15) who have had surgery and long-term post-operative care.

## Patients and Methods

Data from between the opening of the center in April 2007 and December 2019 were collected in a specific database which included essential information on patient characteristics, clinical data as diagnosis, surgical procedure, length of stay, readmissions, follow-up visits, major complications, and redo operations.

Descriptive data were reported as frequencies and percentages, median, mean averages with SD, depending on the data format and distribution. RHD patients are herein presented overall and into subgroups, divided according to the surgical procedure [isolated mitral (MV) or aortic valve (AV) and multiple-valve surgery (MV + TV + AV; MV + TV; MV + AV)]. MV surgery was categorized as repair or replacement procedure. We used the χ2 or Fisher's exact tests to compare categorical variables and the *T*-test and ANOVA or the Mann-Whitney *U* and Kruskal-Wallis tests to compare continuous variables, depending on their distribution and the number of groups.

Mortality was analyzed as early perioperative mortality (in-hospital or within 30 days from operation) and presented with 95% confidence intervals (95% CI).

Survival analysis for 5-year mortality was presented with Kaplan-Meier curves and analyzed using the log-rank test to compare survival in MV repair and replacement surgeries. Univariate and multivariable Cox proportional hazard models were developed to assess the association between surgery type and outcome. Besides age and sex, the variables found to be associated to outcome in the univariate model, were considered as covariates in the multivariable one. The adjusted hazard ratio estimate (HR) is presented with 95% CI. *P* ≤ 0.05 was considered statistically significant; SAS 9.4 software (SAS Institute Inc., Cary, North Carolina, USA) was used for all analyses.

## Results

In the time interval 2007–2019, 8,369 patients had open-heart surgery at the center, and out of this overall number, 2,354 were children, mainly from Sudan (74.6% overall and 78% for RHD patients) but with 598 and 279, respectively, admitted from other countries through the Regional Program (Supplementary Figure 1; Figure 1). The majority (1,318, 56%) of these children suffered from RHD, while 1,025 (43.5%) underwent surgery for CHD and 11 (0.5%) for other reasons (Supplementary Figure 2). Children with RHD are further analyzed: 612 (46.4%) were operated for isolated MV, 73 (5.5%) for isolated AV, and 633 (48.0%) for combined mitral + tricuspid and/or aortic valve disease. Table 1 reports the main characteristics of the 1,318 RHD children with a mean age of 11 years (SD = 2.4), mainly from Sudan (78.8%). Early mortality was 47/1,318 (3.6%, 95% CI 2.6–4.7), including children with urgent, unplanned admission and reoperations who showed higher mortality rates (6.0, 95% CI 3.0–10.4 and 7.0% 95% CI 3.4–12.5, respectively). No marked differences in mortality was found when reoperations occurred in the first 6 months after first surgery were compared to those occurred later, with rates of 9% (*n* = 5/55) and 6% (*n* = 5/87), respectively (data not shown in table). As expected, children needing multiple valve surgery underwent urgent operations significantly more often and had worse outcomes. Mortality rates were comparable over time, with values of 4.4% (*n* = 22/499), 2.7% (*n* = 11/405) and 3.3% (*n* = 14/414), in the periods 2007–2011, 2012–2015, and 2016–2019, respectively (data not shown in table). The overall 5-year survival was 85.0% (95% CI 82.7–87.3), with 150 deaths and a sustainable proportion lost to follow-up (21%), which was equally distributed across subgroups.

**Table 1:** Main characteristics and clinical outcomes of the overall sample of 1,318 RHD children and stratified according to valve surgery subgroups: isolated mitral valve (MV), isolated aortic valve (AV), and mitral and tricuspid and/or aortic valves (MV + TV and/or AV).

**Patients demographic and clinical characteristics**		**Total**	**MV (*n* = 1,318)**	**AV**	**Mitral+TV and/or AV**	***p*-value^a^**
		**(*n* = 1,318)**	**(*n* = 612, 46.4%)**	**(*n* = 73, 5.5%)**	**(*n* = 633, 48.0%)**	
Country: Sudan,	*n* (%)	1,039 (78.8)	459 (75.0)	66 (90.4)	514 (81.2)	0.001
Females,	*n* (%)	672 (51.0)	361 (59.0)	15 (20.5)	296 (46.8)	<0.001
Age,	Median, Mean (SD^b^)	12.0, 11.1 (2.4)	11.0, 10.8 (2.6)	12.0, 11.9 (1.7)	12.0, 11.4 (2.2)	<0.001
BMI,	Median, Mean (SD^b^)	13.2, 13.5 (2.5)	13.0, 13.5 (2.9)	14.4, 15.0 (2.3)	13.2, 13.3 (2.0)	<0.001
Urgency,	*n* (%)	185 (14.0)	66 (10.8)	4 (5.5)	115 (18.2)	<0.001
Reoperation,	*n* (%)	143 (10.9)	75 (12.2)	5 (6.8)	63 (10.0)	0.242
Months to reoperation,	Median, Mean (SD^b^)	14.5, 24.3 (28.3)	15, 25.2 (29.9)	17, 28.0 (31.2)	12, 22.8 (26.4)	0.289
<6	*n* (%)	52 (36.6)	30 (40.0)	1 (20.0)	21 (33.9)	0.578
6–24	*n* (%)	40 (28.2)	17 (22.7)	2 (40.0)	21 (33.9)	
>24	*n* (%)	50 (35.2)	28 (37.3)	2 (40.0)	20 (32.3)	
In-hospital or 30-days mortality,	*n*, % [95%CI]	47, 3.6 [2.6–4.7]	17, 2.8 [1.6–4.4]	0, 0.0 [0.0–4.9]^c^	30, 4.7 [3.2–6.7]	0.039
In urgent surgeries	*n*, % [95%CI]	11, 6.0 [3.0–10.4]	3, 4.6 [1.0–12.7]	0, 0.0 [0.0–60.0]^c^	8, 7.0 [3.1–13.3]	0.803
In reoperated patients	*n*, % [95%CI]	10, 7.0 [3.4–12.5]	6, 8.0 [3.0–16.6]	0, 0.0 [0.0–52.3]^c^	4, 6.3 [1.8–15.5]	0.830

*EMERGENCY-NGO Salam Centre, Khartoum 2007–2019*.

a *Between groups comparison: χ^2^ test or Fisher exact test, and or ANOVA or Kruskal-Wallis test, depending on variable distribution*.

b *SD, Standard Deviation*.

c *One-sided 97.5% confidence interval*.

**Figure 2:**
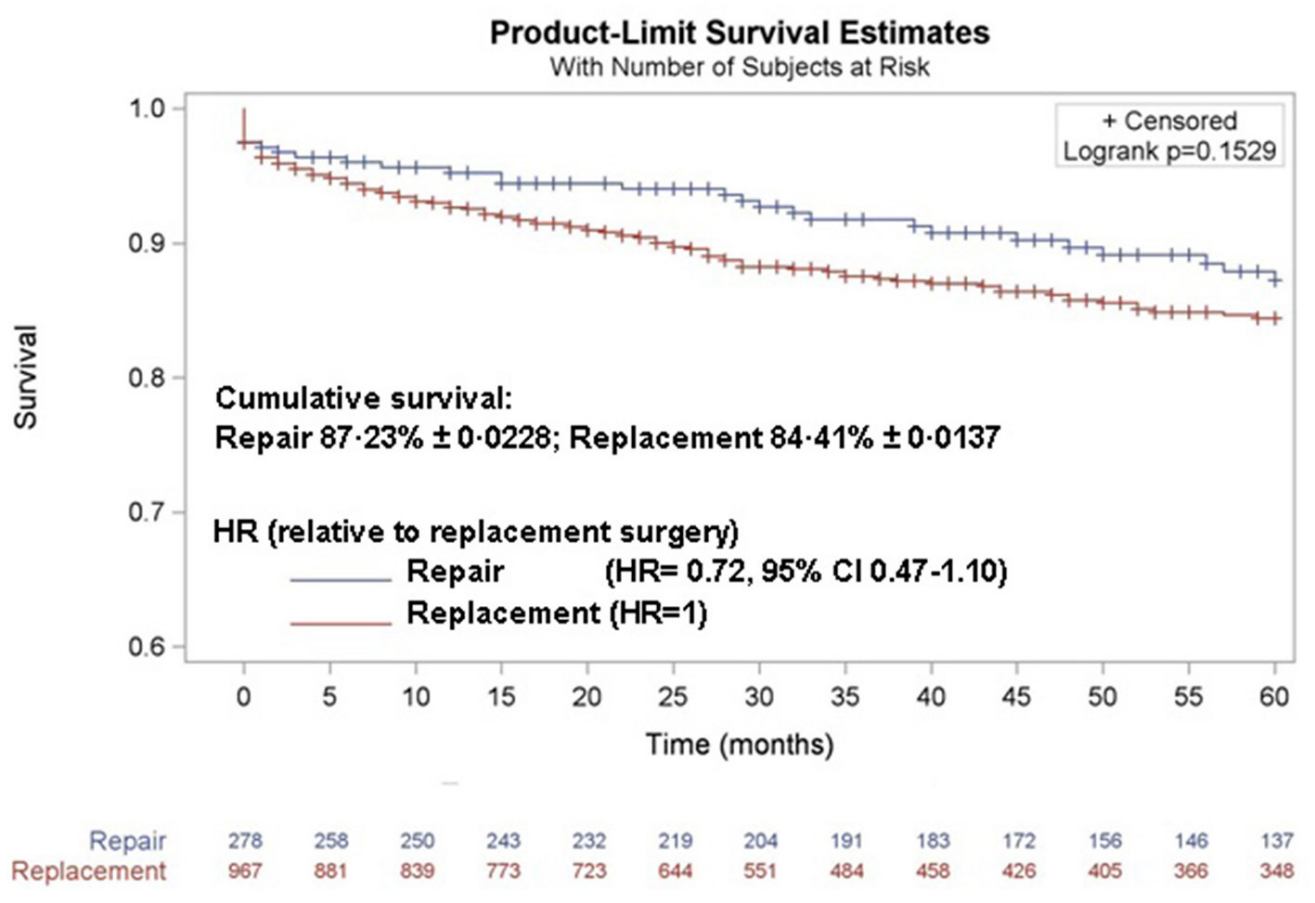
Five-year survival for the MV pediatric population according to surgical type of procedure: replacement with mechanical valve or repair of the damaged valve.

The outcomes were not significantly different when surgical approaches (repair of the mitral valve or replacement with mechanical valve) were compared. Although without statistical significance, outcomes were better in children undergoing MV repair, both as early mortality (2.5% for repair vs. 4.1% for replacement, Table 2) and as 5-year mortality (87.2 and 84.4%, respectively, Figure 2). This result was confirmed even when age, sex, urgency, multiple-valve involvement, and reoperation (entered as a time-depending covariate), were included in Cox proportional hazard model as potential confounders (HR = 0.72, 95% CI 0.47–1.10, for repair surgery).

**Table 2:** Main characteristics and clinical outcomes of the overall sample of 1,245 MV or (MV + TV and/or AV) children, and for repair and replacement surgery separately.

**Patients demographic and clinical characteristics**		**Total (*n*= 1,245)**	**Repair (*n* = 278, 22.3%)**	**Replacement (*n* = 967, 77.7%)**	***p*-value^a^**
Country: Sudan,	*n* (%)	973 (78.2)	229 (82.4)	744 (76.9)	0.053
Females,	*n* (%)	657 (52.8)	158 (56.8)	499 (51.6)	0.124
Age,	Median, Mean (SD^b^)	12.0, 11.1 (2.5)	11.0, 10.5 (2.7)	12.0, 11.2 (2.4)	<0.001
BMI,	Median, Mean (SD^b^)	13.1, 13.4 (2.5)	13.2, 13.5 (2.1)	13.1, 13.4 (2.6)	0.710
Urgency,	*n* (%)	181 (14.5)	28 (10.1)	153 (15.8)	0.015
Reoperation,	*n* (%)	138 (11.1)	62 (22.3)	76 (7.9)	<0.001
Months to reoperation,	Median, Mean (SD^b^)	13.5, 24.0 (28.2)	12.0, 23.4 (29.2)	14.0, 24.5 (27.6)	0.290
<6	*n* (%)	51 (37.2)	25 (40.3)	26 (34.7)	0.784
6–24	*n* (%)	38 (27.7)	16 (25.8)	22 (29.3)	
>24	*n* (%)	48 (35.0)	21 (33.9)	27 (36.0)	
In-hospital or 30-days mortality,	(*n*/Total), % [95%CI]	(47/1,245), 3.8 [2.8–5.0]	(7/278), 2.5 [1.0–5.1]	(40/967), 4.1 [3.0–5.6]	0.283
In urgent surgeries	(*n*/Total), % [95%CI]	(11/181), 6.1 [3.1–10.6]	(1/28), 3.6 [0.1–18.4]	(10/153), 6.5 [3.2–11.7]	0.999
In reoperated patients	(*n*/Total), % [95%CI]	(10/138), 7.3 [3.5–12.9]	(2/62), 3.2 [0.4–11.2]	(8/76), 10.5 [4.7–19.7]	0.185

*EMERGENCY-NGO Salam Centre, Khartoum 2007–2019*.

a *Between groups comparison: χ^2^ test or Fisher exact test, and or T-test or Wilcoxon test, depending on variable distribution*.

b *SD, Standard Deviation*.

## Discussion

Severe and advanced RHD in children is frequent in the SSA and nearly half of the children treated at the *Salam* Centre needed multiple valve surgery and 14.5% had an unscheduled, urgent admission and operation because of no more treatable cardiac failure. The overall 3.8% [95% CI 2.8–5.0] in hospital/30 days mortality is higher than the mortality reported by others in pediatric patients, which is 2.2% in India (19) and 1.7% in Malaysia (20). Similarly the overall 5-year survival (87.2 and 84.4%, for repair and replace procedures, respectively) is lower than the 95.2 and 93.9% reported by these authors. Comparisons are difficult but one evident difference between these series, is the surgical approach with a much higher percentage of repair procedures, 100% (19) and 80.2% (20) vs. 22.3% in our experience. It is commonly believed that repair surgery has lower early mortality and in absence of long-term anticoagulation, lower mortality from thrombosis and bleeding. In the opinion of our surgeons, however, very advanced valve disease cannot be cured by repair, and a different patient selection in the three institutions is therefore a possible explanation. The survival curves reported in Figure 2, suggest a trend to a better outcome for patients who undergo repair procedures, but the risk for valve replacement remains without statistical significance (HR = 0.72, 95% CI 0.47–1.10, for repair surgery) also considering potential confounders. This is, in our opinion, a good quality indicator for our long-term post-operative care and particularly the anticoagulant treatment, which presents particular difficulties in children and adolescents.

The proportion of available long-term follow-up data was in line with the recommended thresholds of 60–80% to avoid major selection bias related to loss to follow-up (21, 22). Moreover, 21% of patients lost at long-term follow-up is not too high if we consider that some patients may do well and are not on anticoagulants, other may be in follow-up in other institutions abroad; some probably died from acute cardiac complications or from one of the many other possible causes, as traffic accidents, infectious diseases and so on. Completing data on patients lost at follow-up as well as causes of death is nearly impossible.

Children with RHD are an indicator of a poor socio-economic condition and an inadequate health system, which clearly will not be cured by cardiac surgery. Building a hospital, delivering cardiac surgery free of charge, and offering long-term post-operative care may help to foster belief in the possibility of positive change in the future.

### Strengths and Limitations of This Article

The importance of this article is to inform the medical community about this extraordinary project. The long-running work at the *Salam* Centre for Cardiac Surgery offers a model of humanitarian surgery that is different from many other approaches: affordability, continuity, accessibility, high-quality medicine and lack of fees are the key factors in its success, together with the vital involvement and participation of Sudanese authorities and institutions.

The main limitations are intrinsic to the nature of a retrospective cohort study over many years. We wanted to include all patients operated on, starting from the first one in 2007, but obviously the database changed from one with very essential information (demographics, the surgical procedure, and hospital outcome) to a much more comprehensive one, with clinical and ultrasound data linked to the outpatient and anticoagulant clinic databases. For this reason, more detailed analysis of the clinical data is not possible for the whole population.

## Data Availability Statement

The data analyzed in this study is subject to the following licenses/restrictions: The de-identified data of patients that underlie the results reported in this article (text, Tables 1, 2, Figures 1, 2, and Supplementary Material) and a data dictionary defining each field in the set will be shared. Data will be available 2 months after publication for the subsequent 5 years to researchers, who will provide a methodologically sound proposal. Proposals should be directed to lorenzo.valgoi@emergency.it. To gain access, data requestors will be asked to sign a data access agreement. Requests to access these datasets should be directed to Lorenzo Valgoi, lorenzo.valgoi@emergency.it.

## Ethics Statement

The studies involving human participants were reviewed and approved by Comitato Etico Università degli Studi di Milano, Milan, Italy. Written informed consent from the participants' legal guardian/next of kin was not required to participate in this study in accordance with the national legislation and the institutional requirements.

## Author Contributions

RM contributed to the study's concept, writing, and supervising the structure of the paper. MQ collected data and interpreted the results. LV constructed and managed the database. LC performed the analysis and contributed to writing. SL contributed to data acquisition, the study's concept, and interpretation of the findings. EG contributed to data collection and revision of the document. LR managed the Regional Program and contributed to writing. NE managed the oral anticoagulant clinic and contributed to writing. SG contributed to writing and coordinated the working group. DR contributed as a perfusionist to the activity of the operating room. MS critically revised the manuscript. AS contributed to data acquisition, the study's concept, and interpretation of the findings. ML and GP contributed to the study's concept and organization, design, data interpretation, and writing. GS revised the text. All authors approved the final manuscript and share the responsibility for the decision to submit for publication.

## Conflict of Interest

The authors declare that the research was conducted in the absence of any commercial or financial relationships that could be construed as a potential conflict of interest.

## Publisher's Note

All claims expressed in this article are solely those of the authors and do not necessarily represent those of their affiliated organizations, or those of the publisher, the editors and the reviewers. Any product that may be evaluated in this article, or claim that may be made by its manufacturer, is not guaranteed or endorsed by the publisher.

## Supplementary Material

Supplementary Figure 1 Study flow chart.

Supplementary Figure 2 Year-by-year numbers of operated children (under 15), divided by disease.

Supplementary Table 1 The *Salam* Centre Working Group (2007–2020).
