# Supplementary figures and images for: Treating Children With Advanced Rheumatic Heart Disease in Sub-Saharan Africa: The NGO EMERGENCY's Project at the Salam Centre for Cardiac Surgery in Sudan
Source: Front Pediatr. 2021 Aug 20;9:704729. doi: 10.3389/fped.2021.704729 (PMC8417837; doi:10.3389/fped.2021.704729)

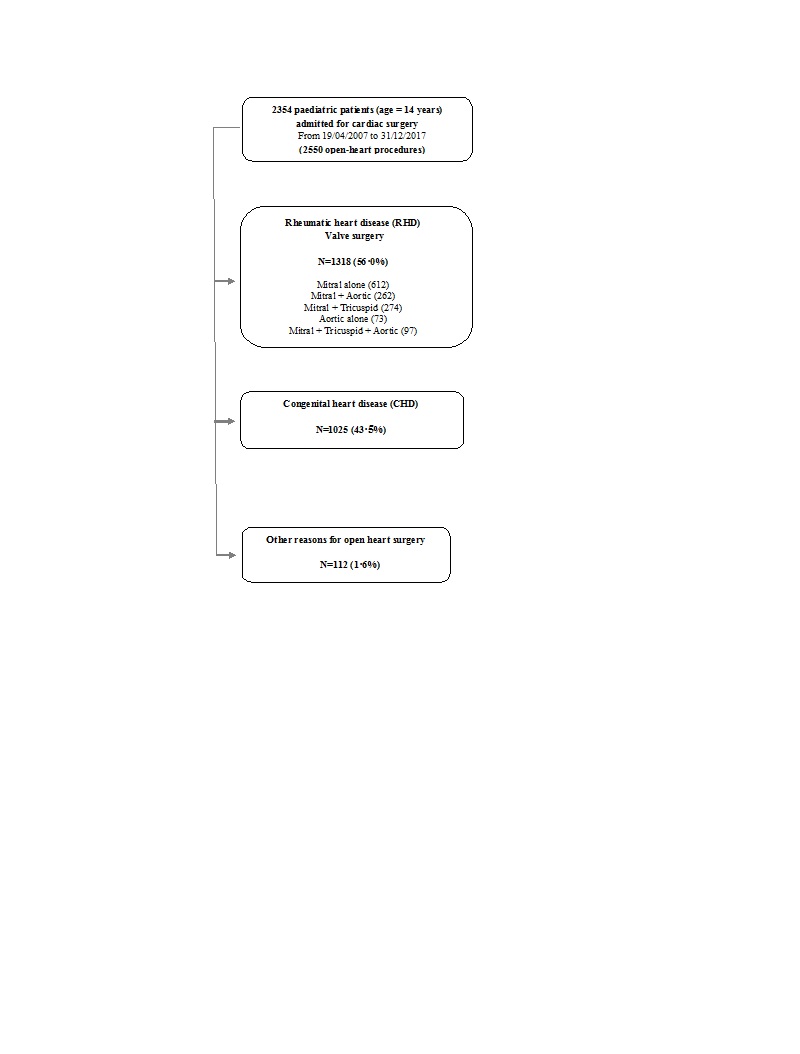

Supplement: Supplementary Figure 1 — Study flow chart. [file Image_1.JPEG]

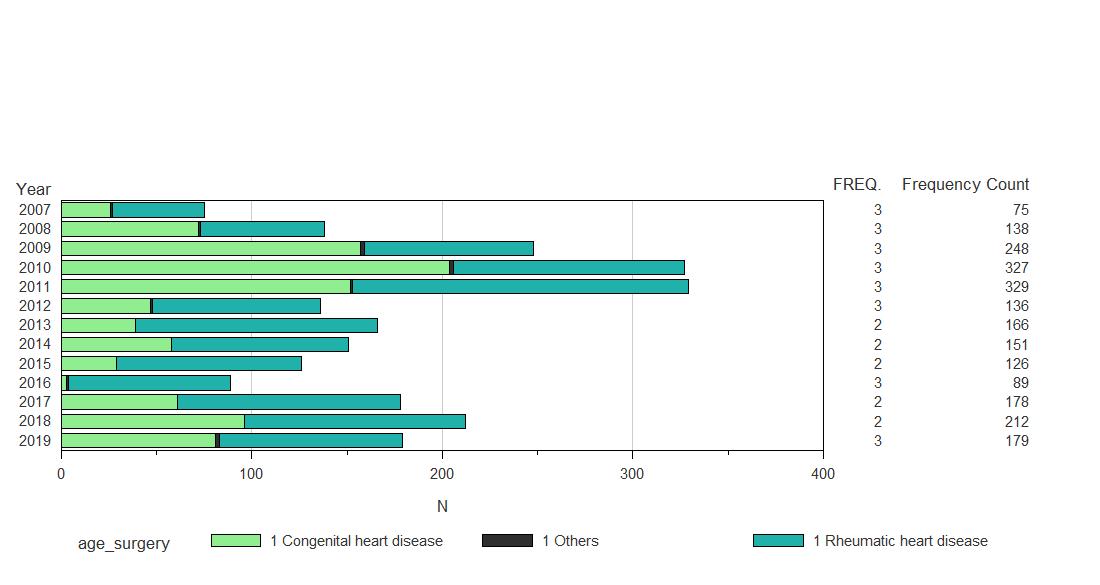

Supplement: Supplementary Figure 2 — Year-by-year numbers of operated children (under 15), divided by disease. [file Image_2.JPEG]
