# Supplementary material for: Treating Children With Advanced Rheumatic Heart Disease in Sub-Saharan Africa: The NGO EMERGENCY's Project at the Salam Centre for Cardiac Surgery in Sudan
Source: Front Pediatr. 2021 Aug 20;9:704729. doi: 10.3389/fped.2021.704729 (PMC8417837; doi:10.3389/fped.2021.704729)
Supplement: Supplementary Table 1 — The Salam Centre Working Group (2007–2020). [file Table_1.DOCX]

# Table S.1 The SALAM Centre Working Group (2007-2020)

| **The SALAM Centre Working Group (2007-2020)** |  |
| --- | --- |
| Abbruzzese Piero | Surgeon |
| Abeer Agos Golda | Nurse |
| Al Radia Sarf Aldeen | Nurse |
| Amà Ruggero | Anaesthetist |
| Amel Hashim Abdel Elbien | Nurse |
| Ammirata Simona | Nurse |
| Anastasio Raffaela | Anesthesia |
| Andric Vladimir | Internal Doctor |
| Anning Lucy | Nurse |
| Areeg Moawia Alrasheed | Nurse |
| Asta Angiolino | Surgeon |
| Baraldi Caterina | Anestesiologist |
| Barbieri Andrea | Cardiologist |
| Barbieri Daniela | Cardiologist |
| Bates Rachel | Nurse |
| Becciu Giampietro | Nurse |
| Benetti Valentina | Cardiologist |
| Bentivegna Giovanni | Cardiologist |
| Berghimani Afra | Nurse |
| Berti Marina | Laboratory Doctor |
| Bianco Nicola | Anesthetist |
| Bini Margherita | Cardiologist |
| Boldini Alessandra | Cardiologist |
| Bonetti Francesca | Nurse |
| Bortalato Chiara | Anestesiologist |
| Bozinovska Marja | Anesthesist |
| Brogiato Giorgio | Nurse |
| Bruletti Alessandra | Nurse |
| Buffa Giuseppina | Pharmacist |
| Buono Gabriella | Anaesthetist |
| Busi Marzio | Surgeon |
| Buzzetti Serena | Nurse |
| Calissano Luca | Nurse |
| Calza Stefania | Radiologist |
| Cammarata Silvia | Pharmacist |
| Canestrelli Giuseppa | Anesthetist |
| Canu Antonella | Cardiologist |
| Caporuscio Luciana | Nurse |
| Caputo Enrico | Surgeon |
| Cardu Gabriele | Anesthetist |
| Carena Laura | Nurse |
| Carraro Giuseppe | Biomedical Engeneer |
| Cascia Vito | Perfusionist |
| Castillo Cristian | Cardiologist |
| Castracane Walter | Anesthesit |
| Casula Margherita | Cardiologist |
| Cattaneo Sara | Nurse |
| Centamore Guido | Cardiologist |
| Centofantipaolo | Surgeon |
| Cerruto Giuliana | Cardiologist |
| Chacon Daniela | Pharmacist |
| Chironi Elisa | Anestesiologist |
| Cini Roberto | Cardiac Surgeon |
| Cipriani Flora | Anesthesit |
| Claudio Praiola | Cardio Surgeon |
| Coco Rovena | Laboratory Doctor |
| Collareta Michele | Anesthetist |
| Comis Marco | Anestesiologist |
| Confortini Luca | Nurse |
| Conte Antonia | Nurse |
| Cormio Manuela | Anestesiologist |
| Cottini Michela | Cardiologist |
| Crestan Roberto | Hospital Manager |
| Cristante Alessia | Anaesthetist |
| D'amato Roberto | It System Administrator |
| D'amico Chiara | Perfusionist |
| D'amico Giovanna | Anesthetist |
| Damir Fredinger | Nurse |
| D'anna Margherita | Anesthetist |
| De Costanzi Elena | Nurse |
| De Feo Mara | Cardiologist |
| De Lellis Stefano | Physiotherapist |
| De Marzo Simona | Nurse |
| De Serio Daniela | Cardiologist |
| Dekic Marco | Nurse |
| Del Gaudio Eleonora | Regional Coordinator |
| Del Monaco Chiara | Perfusionist |
| Del Pace Stefano | Cardiologist |
| Della Grazia Erica | Cardiologist |
| Delli Guanti Michela | Nurse |
| Di Battista Dafne | Nurse |
| Di Summa Michele | Surgeon |
| Di Tria Marica | Cardiologist |
| Djikanovic Nebojsa | Perfusionist |
| Donolato Piergiorgio | Nurse |
| Dottori Vincenzo | Cardiac Surgeon |
| Dragan Jovanovic | Perfusionist |
| Edimond Elisa | Nurse |
| Ellena Marco | Anesthetist |
| Ena Laura | Nurse |
| Enrico Sponziello | Biomedical Engeneer |
| Fabbiani Tamara | Nurse |
| Facetti Sara | Cardiologist |
| Falchi Marco | Anestesiologist |
| Farini Valentina | Pharmacist |
| Favaro Luigi | Cardiologist |
| Ferlicca Daniela | Anestesiologist |
| Fichet Solange | Nurse |
| Fiorito Valentina | Cardiologist |
| Florescu Maria | Cardiologist |
| Fontana Alessandra | Cardiologist |
| Forni Sauro | Ot Nurse |
| Fracasso Giulia | Anaesthetist |
| Frassani Romeo | Surgeon |
| Gaglio Marco | Anestesiologit |
| Gastaldi Silvana | Nurse |
| Gatti Claudio | Hospital Manager |
| Gehan Hashim Ramadan | Regional Program Officer |
| Gherbi Giordano | Nurse |
| Ghezzo Fabiana | Nurse |
| Giannakopoulou Dimitra | Nurse |
| Gigli Nicoletta | Physiotherapist |
| Giusti Giuliano | Cardiologist |
| Gonzale Rodrigez Raul | Anestesiologist |
| Gordana Cabraja | Nurse |
| Graniero Ascanio | Surgeon |
| Guerzi Sara | Nurse |
| Hamimy Fathy Mohammed Salih | Nurse |
| Hoda Alfadil | Nurse |
| Ilic Nemanja | Nurse |
| Ilic Stojadin | Head Nurse |
| Intili Aldo | Surgeon |
| Iori Davide | Anestesiologist |
| Irace Francesco | Surgeon |
| Javanovich Dragan | Perfusionist |
| Jawaher Hassan Abaker | Nurse |
| Jelena Zlatic | Nurse |
| Josifovski Borko | Cardiologist |
| Jovanov Vancho | Anesthestist |
| Kausal Kishore Tiwari | Surgeon |
| Knezevic Niska | Nurse |
| Kovacevi Nemanja | Nurse |
| Kreuzer Eckart | Surgeon |
| Lamberti Alessandro | Cardiologist |
| Lampugnani Mariano | Nurse |
| Lapolla Fabiola | Anesthetist |
| Lattanzio Maria Angela | Cardiologist |
| Lazarevic Zlatko | Nurse |
| Ledenski Ljubica | Nurse |
| Lindblom Dan | Surgeon |
| Livaja Martina | Nurse |
| Loffi Marco | Cardiologist |
| Maccario Emanuele | It |
| Magda Abdulla Alzain | Nurse |
| Maha Osman | Nurse |
| Malisano Gabriella | Nurse |
| Manahel Bador Saad | Nurse |
| Manar Majzoub Badi | Nurse |
| Mancino Giuseppe | Anesthestist |
| Maraio Lorenzo | Biomedical Engeneer |
| Marchini Maya | Laboratory Doctor |
| Marelli Andrea | Nurse |
| Marinelli Alessandro | Cardiologist |
| Marogna Martina | Nurse |
| Martara Marco | Pharmacist |
| Martella Nunzia | Anestesiologist |
| Martinotta Chiara | Nurse |
| Marwa Kamal Aldin - | Nurse |
| Marzaduri Graziella | Physiotherapist |
| Mashael Mohammed Alamin | Nurse |
| Masini Franco | Cardiologist |
| Masoero Giovanni | Cardiologist |
| Mattarello Lara | Physiotherapist |
| Maurizio Roberto | Surgeon |
| Mazzanti Andrea | Cardiologist |
| Meroni Veronica | Anesthesiologist |
| Michela Delle Guanti | Nurse |
| Milan Stefanovic | Nurse |
| Milete Vanessa | Perfusionist |
| Milleti Filippo | Nurse |
| Mirjana Ilic | Nurse |
| Mirza Becirovic | Cardiologist |
| Missana Massimo | Nurse |
| Mocini Alessandro | Cardiologist |
| Modather Omer Hassan Osman | Nurse |
| Mohannad Abbass Ahmed | Surgeon |
| Mohannad Abdekrahman Mohamed | Surgeon |
| Mona Tag Eldin Farag | Cardiologist |
| Monti Giorgio | General Practicioner |
| Monzo Luca | Cardiologist |
| Morichi Valeria | Internal Doctor |
| Mrdjic Ljubica | Nurse |
| Mwada Hassan Awad | Nurse |
| Nabiha Hasabelrasool | Nurse |
| Nada Ahmed Mohamed | Medical Officer |
| Nannini Emanuele | Hospital Manager |
| Napoleone Alessandra | Anesthetist |
| Neri Gianfilippo | Cardiologist |
| Ninci Antonella | Laboratory Doctor |
| Nonini Sandra | Anestesiologist |
| Nosiba Abdalgadir Mohamed | Nurse |
| Ntalarizou Evangelia | Surgeon |
| Nubile Peppino | Laboratory Doctor |
| Olla Marco | Anestesiologist |
| Ombashair Esmail | Nurse |
| Ove Andreas Hagen | Anestesiologist |
| Owens Simon | It Technician |
| Pace Carlo | Surgeon |
| Pacher Valentina | Cardiologist |
| Pacilli Pina | Cardiologist |
| Parenzan Luciano | Surgeon |
| Parrino Pietro | Field Operations Director |
| Pedroni Giulia | Nurse |
| Pessina Carla | Anestesiologist |
| Placek Ondrej | Perfusionist |
| Pop Cristina | Radiologist Technician |
| Portolan Mario | Anesthesiologist |
| Predja Ognjen | Hospital Manager |
| Puglisi Flavio | Laboratory Doctor |
| Pula Giorgio | Anesthesiologist |
| Puntila Juha | Surgeon |
| Putignano Giuseppe | Cardiologist |
| Quattrochiocchi Maria Luigia | Cardiologist |
| Radaelli Sofia | Cardiologist |
| Rasha Ibrahim | Nurse |
| Raucci Rosa | Cardiologist |
| Redighieri Sara | Nurse |
| Reem Abd Alati Salih | Nurse |
| Reham Abdelghafar Abdelkhair | Nurse |
| Reyes Eva | Cardiologist |
| Risica Gabriele | Cardiologist |
| Rocchi Daniela | Perfusionist |
| Rolla Luca | Nurse |
| Rossi Cristina | Nurse |
| Ruzic Tamara | Nurse |
| Saccani Stefano | Surgeon |
| Saccavino Erica | Anesthetist |
| Sahwa Abdulla Ali | Medical Officer |
| Samar Mohamed Ali | Nurse |
| Sanaa Yousif Ahmed | Nurse |
| Santandrea Chiara | Nurse |
| Santoro Francesco | Cardiac Surgeon |
| Sarzi Braga Simona | Cardiologist |
| Saso Stoilov | Perfusionist |
| Scapaticci Margherita | Laboratory Doctor |
| Schneeberger Eric William | Surgeon |
| Serra Massimo | Surgeon |
| Shemaa Mohamed Salih | Nurse |
| Slaveski Dimce | Anaesthetist |
| Solbiati Arturo | Laboratory Doctor |
| Sowsan Sayed Abdelgafar | Nurse |
| Spanò Antonella | Nurse |
| Sreckovic Nena | Nurse |
| Sulpizio Jasmine | Anaesthetist |
| Sundic Jasna | Nurse |
| Tedeschi Rossella | Anaesthetist |
| Testa Filippo | Anesthesist |
| Torri Adriano | Nurse |
| Torta Romina | Ot Nurse |
| Tortu Anonietta | General Practicioner |
| Trajkovsky Peter | Anesthesist |
| Tromba Andrea | Perfusionist |
| Turchi Elisabetta | Cardiologist |
| Vescovini Silvia | Perfusionist |
| Vigni Elisa | Nurse |
| Villa Livio | Anaesthetist |
| Vitali Ettore | Cardio Surgeon |
| Vojnic Marina | Nurse |
| Vullo Carmelo | Anaesthetist |
| Wala Abdelrahman Alazeim Fadalmola | Nurse |
| Williams Martina | Nurse |
| Zanotti Nadia | Nurse |
| Zoppi Nicola | Engeener |
